# The association between obesity-related indicators and female infertility: the United States National Health and Nutrition Examination Survey, 2013–2018

**DOI:** 10.3389/fendo.2025.1588965

**Published:** 2025-06-23

**Authors:** Chunming Yu, Haishuang Wu, Xin Sun, Min Cao, Jing Yuan

**Affiliations:** Department of Obstetrics and Gynecology, The First Affiliated Hospital of Harbin Medical University, Harbin Medical University, Harbin, Heilongjiang, China

**Keywords:** obesity, infertility, National Health and Nutrition Examination Survey, restricted cubic splines analysis, receiver operating characteristic analysis

## Abstract

**Background:**

Infertility is a public health issue closely related to obesity. However, the relationship between obesity-related indicators and infertility is currently uncertain. The present study aimed to explore the association between obesity-related indicators and female infertility.

**Methods:**

This cross-sectional study included data for 2,875 adult females aged 20–45 years from the National Health and Nutrition Examination Survey conducted between 2013 and 2018. Logistic regression models, restricted cubic spline (RCS), and receiver operating characteristic (ROC) analyses were used to evaluate the relationship between obesity-related indicators [body shape index (ABSI), weight-adjusted waist index (WWI), body roundness index (BRI), waist-to-height ratio (WHtR), non-HDL cholesterol to HDL cholesterol ratio (NHHR), relative fat mass (RFM), body mass index (BMI), and waist circumference (WC)] and female infertility.

**Results:**

Adult females were divided into five groups based on their ABSI, WWI, BRI, WHtR, NHHR, RFM, BMI, and WC. Individuals in the highest quintile for ABSI, WWI, BRI, WHtR, NHHR, RFM, BMI, and WC had a higher risk of infertility compared to those in the lowest quintile. The respective adjusted odds ratio values were 1.65 (95% confidence interval (CI), 1.14 to 2.42), 1.71 (95% CI, 1.15 to 2.57), 2.09 (95% CI, 1.39 to 3.19), 2.09 ( 95% CI, 1.39 to 3.19), 1.71 (95% CI, 1.14 to 2.59), 2.09 (95% CI, 1.39 to 3.19), 2.10 (95% CI, 1.40 to 3.18), and 2.28 (95% CI, 1.52 to 3.47). The *p* for trend values were 0.027, <0.001, <0.001, <0.001, 0.002, <0.001, <0.001, and <0.001after controlling for a series of confounding factors. RCS analyses showed a linear correlation between ABSI, WWI, BRI, WHtR, RFM, BMI, and WC and infertility (*P*_nonlinear_ > 0.05). A nonlinear association was observed between NHHR and infertility (*P*_nonlinear_ = 0.006). The ROC curve demonstrated that BRI, WHtR, RFM, and WC exhibited relatively high diagnostic efficiency for infertility, particularly among women aged 20–35 years.

**Conclusions:**

The WHtR, RFM, WC, and BRI are superior to BMI in predicting and diagnosing infertility, particularly among individuals aged 20–35 years. Consequently, these indices show promise as more effective tools for identifying populations at an early risk of infertility. To confirm these findings, future studies, such as Mendelian randomization or cohort studies, are warranted.

## 1 Introduction

Infertility is a universal health issue that is defined as a failure to establish a clinical pregnancy after one year of regular, unprotected sexual intercourse. It affects approximately 10% of reproductive-aged couples attempting to conceive (1, 2). Infertility is estimated to have an impact on as many as 186 million people worldwide (3). It has been reported that couples in developed countries suffer from primary infertility more often (4). Although male infertility accounts for more than half of the cases, infertility remains a serious social burden on women (3). Identifying potential risk factors that influence fertility prevention and management, as well as reliable markers for predicting infertility risk, holds great significance for public health.

Obesity is defined as having a body mass index (BMI) of 30 kg/m² or above. It significantly increases the risk of cardiovascular disease (CVD), type 2 diabetes mellitus (T2DM) (5), metabolic disorders, cancers (6), and infertility (7). BMI is the most commonly used body mass indicator and is closely related to various diseases associated with excessive weight and obesity. Abdominal obesity is characterized by the accumulation of fat around the visceral organs within the abdominal cavity. It is a widespread issue that is closely linked to infertility (8). When assessing the degree of obesity, BMI does not take into account fat distribution, particularly in cases of abdominal obesity. Therefore, there may be limitations in using BMI to predict the occurrence of infertility (9). However, the relationship between other obesity indicators, particularly those related to abdominal obesity, and infertility remains unclear.

New anthropometric indices that combine height and waist circumference (WC), such as body shape index (ABSI), weight-adjusted waist index (WWI), body roundness index (BRI), or waist-to-height ratio (WHtR), can effectively provide detailed information about body shape and fat distribution. In addition, the non-HDL cholesterol to HDL cholesterol ratio (NHHR) and relative fat mass (RFM) can reflect the status of visceral fat.

On this basis, the present study for the first time utilized data from the National Health and Nutrition Examination Survey (NHANES) to systematically investigate the relationship between indicators associated with fat distribution and infertility issues and separately examined their predictive power for infertility. This information may be crucial for enhancing infertility diagnosis and prevention, as well as promoting the development of reproductive health.

## 2 Methods

### 2.1 Study population

The NHANES is a cross-sectional study utilizing a stratified, multistage sampling design, with data for the United States civilian non-institutionalized population released in two-year cycles. The program covers clinical, physical, and laboratory examinations, as well as interviews in order to obtain diet and health indicators. It has played a pivotal role in informing health policy decisions. Detailed NHANES information has been provided previously (10). The NHANES protocol was approved by the National Center for Health Statistics Research Ethics Review Board, and all participants provided informed consent. Data accumulation was performed by the National Center for Health Statistics with approval from their ethics review board. All databases can be obtained from the NHANES website (https://wwwn.cdc.gov/nchs/nhanes/Default.aspx).

A total of 29,400 participants in the NHANES (2013–2018) were evaluated for the present study. The research sample excluded male participants (n=14,452), those younger than 20 years or older than 45 years (n=11,093), participants with missing information on infertility (n=603), those who have undergone hysterectomy or bilateral oophorectomy (n=141), and individuals with missing critical baseline information, such as height and weight (n=236). As a result, 2,875 adult females were enrolled in the study, including 369 with infertility issues.

### 2.2 ABSI, WWI, BRI, WHtR, NHHR, and RFM assessment

Anthropometric measurements, such as body height, body weight, and WC, were collected by trained examiners at a mobile examination center equipped with standardized tools. Participants’ body mass was evaluated using calibrated platform scales with a precision of 0.1 kg, and their height was measured with stadiometers while standing and was accurate to 0.1 cm. These measurements were obtained with participants wearing light clothing and no shoes. ABSI was calculated using the following formula: 1000×WC (m) × height (m)^5/6^×weight (kg)^-2/3^ (11). WWI was determined by dividing WC (cm) by the square root of body weight (kg) (12). BRI was calculated using the following formula: 364.2−365.5 × (1−[WC(m)/2 × π]^2^ /[0.5×height(m)] × 2)^1/2^ (13). WHtR was calculated by dividing WC (cm) by the participant’s height (cm) (14). The formula for calculating RFM was as follows: RFM = 64 − (20 × height (cm)/WC (cm)) + (12 × gender), where genders 1 and 0 denoted females and males, respectively (15). The data source for the NHHR calculations was derived from the laboratory data in NHANES called ‘HDL.Doc’ that provides HDL data and ‘TCHOL.Doc’ that provides total cholesterol data. The NHHR data were obtained using the formula for total cholesterol minus HDL and then divided by HDL (16). BMI was calculated as follows: body weight (kg)/height(m)^2^ (17).

### 2.3 Infertility diagnosis

Infertility was defined as a failure to achieve pregnancy after one year of unprotected intercourse. The presence of infertility was determined by self-reporting in a questionnaire, with female participants indicating a positive response to either of the following two questions: “Have you attempted to conceive for at least one year without success?” or “Have you sought medical assistance for infertility?” being classified as ever infertile (18).

### 2.4 Confounding measurements

Potential covariates in the study included age (years), race (Mexican American, Other Hispanic, Non-Hispanic White, Non-Hispanic Black, or Other Race), education level (<9th grade, 9–11th grade, high school graduate, GED or equivalent, some college or associate’s degree, or college graduate or above), smoking status (never smoked, current smoker, or former smoker), regular exercise (yes/no), marital status (married, widowed, divorced, separated, or never married), annual household income (≤$20,000 or>$20,000), alcohol intake (drinks/week), total energy intake (kcal/day), alternative healthy eating index (AEHI),T2DM status (yes/no), cancer status (yes/no), CVD status (yes/no), systemic immune-inflammation index (SII), systolic blood pressure (mmHg), and diastolic blood pressure (mmHg). The amount of alcohol consumed was measured by the number of drinks, where a standard drink was any drink that contained about 0.6 fluid ounces or 14g of pure alcohol. T2DM was defined by a self-reported diagnosis, an HbA1c level of ≥ 6.5%, or a fasting plasma glucose level of ≥7.0mmol/L. CVD was defined as a self-reported diagnosis history of heart failure, coronary heart disease, angina/angina pectoris, heart attack, or stroke (19). The AHEI was developed from the original Healthy Eating Index, which included 11 food components identified through a comprehensive review of studies (14). The SII level was determined by multiplying the platelet count by the neutrophil count/lymphocyte count (20).

### 2.5 Statistical methods

All analyses incorporated dietary sample weights, stratification, and clustering of the complex sampling design to ensure nationally representative estimates according to the NHANES analytic guidelines. General linear models and chi-square tests were used to compare baseline characteristics by quintiles. Continuous variables were expressed as mean ± standard deviation, while classified variables were expressed as percentages. Missing covariables at <5% were filled in using multiple interpolation. When the missing value of a variable was >5%, it was deleted to avoid affecting the results. A two-sided *P* value of <0.05 was considered statistically significant. All statistical analyses were performed using R software version 3.5.3.

#### 2.5.1 Logistic regression models

Adult females were divided into five groups based on their ABSI, WWI, BRI, WHtR, NHHR, RFM, BMI, and WC. Logistic regression models were used to evaluate ABSI, WWI, BRI, WHtR, NHHR, RFM, BMI, and WC and the risk of infertility. Odds ratio (OR) and their 95% confidence interval (CI) were estimated in logistic regression models with the lowest quintile of ABSI, WWI, BRI, WHtR, NHHR, RFM, BMI, and WC as the reference category. A series of potential confounders were adjusted for in the process of statistical analysis, including age, race, education level, smoking status, regular exercise, marital status, annual household income, alcohol intake, total energy intake, AEHI, T2DM status, cancer status, CVD status, SII, systolic blood pressure, and diastolic blood pressure.

#### 2.5.2 Restricted cubic splines analysis

To account for the dose-response relationship (linear or nonlinear) between ABSI, WWI, BRI, WHtR, NHHR, RFM, BMI, and WC and infertility, RCS analyses adjusted for the same variables as the above analyses were performed at the 5th, 50th, and 95th percentiles of the ABSI, WWI, BRI, WHtR, NHHR, RFM, BMI, and WC distributions. Three nodes were set to exclude the most extreme 5% values to reduce the potential impact of the outliers. Nonlinearity tests were performed using the likelihood ratio test.

#### 2.5.3 Receiver operating characteristic curves

ROC curves were used for diagnostic value analysis. The area under the curve (AUC) as measured by the C-statistic was computed to quantify the predictive power of ABSI, WWI, BRI, WHtR, NHHR, RFM, BMI, and WC for infertility.

#### 2.5.4 Sensitivity analysis

Three sets of sensitivity analyses were conducted to verify the stability of the research results. First, some previous research studies determined infertility based on the response to the following question: “Have you ever attempted to become pregnant over at least a year, without becoming pregnant?” Women answering "yes" were considered infertile, whereas those answering "no" were deemed normal (21). Therefore, the present study conducted analysis using only this single question as the criterion for determining infertility in sensitivity analysis 1. Second, in sensitivity analyses 2 and 3, the statistical evaluations were conducted separately within the two age groups of 20–35- and 36–45-year-olds.

## 3 Results

### 3.1 Demographic characteristics of participants

The present study evaluated 2,875 adult females, including 369 cases of infertility. Baseline population characteristics in terms of BRI in quintiles are shown in **Table 1**. Age, race, education level, smoking status, moderate physical activity, marital status, annual household income, AEHI, T2DM status, cancer status, SII, systolic blood pressure, and diastolic blood pressure were significantly different across quintiles 1–5 (*p*<0.05). There was no significant difference in CVD status, alcohol intake, and total energy intake among these quintiles (*p*>0.05).

**Table 1:** Baseline characteristics in terms of quintiles of BRI: NHANES, 2013-2018 (N=2,875).

Characteristics	Q1 (N=575)	Q2 (N=575)	Q3 (N=575)	Q4 (N=575)	Q5 (N=575)	*P* value
Age (years)	29.77 (7.18)	32.39 (7.69)	32.89 (7.22)	33.53 (7.35)	33.74 (7.26)	<0.001
College graduate or above [n, (%)]	245 (0.43)	206 (0.36)	149 (0.26)	103 (0.18)	95 (0.17)	<0.001
Non-Hispanic white [n, (%)]	108 (0.19)	96 (0.17)	113 (0.20)	131 (0.23)	160 (0.28)	<0.001
Current no-smoking [n, (%)]	423 (0.74)	430 (0.75)	418 (0.73)	421 (0.73)	361 (0.63)	<0.001
Regular exercise [n, (%)]	302 (0.53)	294 (0.51)	249 (0.43)	249 (0.43)	239 (0.42)	<0.001
Alcohol intake (drinks/week)	4.04 (41.6)	2.31 (1.63)	2.3 (1.59)	4.29 (41.61)	2.59 (2.12)	0.790
Married [n, (%)]	222 (0.39)	283 (0.49)	253 (0.44)	249 (0.43)	254 (0.44)	<0.001
Total energy (kcal/day)	1922.03 (800.72)	1823.59 (644.51)	1807.84 (630.48)	1854.61 (694.10)	1872.18 (744.5)	0.461
AHEI sore	34.64 (10.00)	34.21 (9.01)	33.92 (8.71)	33.33 (8.23)	31.9 (8.22)	<0.001
SII	484.75 (241.22)	510.3 (288.05)	539.53 (371.18)	579.36 (289.27)	625.01 (306.71)	<0.001
Systolic Blood Pressure (mmHg)	107.97 (9.73)	110.85 (11.63)	111.9 (11.97)	115.66 (13.65)	120.34 (13.8)	<0.001
Diastolic Blood Pressure (mmHg)	66.29 (8.63)	67.42 (10.04)	67.91 (11.10)	69.55 (10.75)	72.42 (11.55)	<0.001
Cancer [n, (%)]	11 (0.02)	14 (0.02)	12 (0.02)	14 (0.02)	19 (0.03)	<0.001
CVD [n, (%)]	7 (0.01)	7 (0.01)	6 (0.01)	10 (0.02)	28 (0.05)	0.595
T2DM [n, (%)]	2 (0)	9 (0.02)	25 (0.04)	45 (0.08)	83 (0.14)	<0.001
BMI (kg/m^2^)	20.7 (1.93)	24.39 (2.25)	28.08 (2.93)	32.8 (3.46)	41.78 (6.82)	<0.001
WC (cm)	74.57 (4.68)	84.12 (4.33)	93.03 (4.83)	103.92 (5.94)	124.15 (13.08)	<0.001
BRI	2.61 (0.41)	3.76 (0.32)	4.95 (0.38)	6.55 (0.56)	9.92 (2.25)	<0.001
RFM	32.14 (2.44)	37.56 (1.21)	41.39 (1.04)	45.05 (1.09)	49.79 (2.19)	<0.001
ABSI	77.63 (3.62)	78.97 (4.08)	79.73 (4.54)	80.36 (4.65)	81.56 (4.53)	<0.001
WWI	10.07 (0.43)	10.57 (0.46)	10.95 (0.50)	11.33 (0.54)	11.94 (0.60)	<0.001
NHHR	1.64 (0.62)	2.01 (0.84)	2.51 (1.13)	2.84 (1.14)	3.07 (1.43)	<0.001
WHtR	0.46 (0.02)	0.52 (0.02)	0.58 (0.02)	0.65 (0.02)	0.77 (0.07)	<0.001

Continuous variables are expressed as mean (SD); Categorical variables are expressed as n (%); Generalized linear models and χ2 test were used to probe for differences in continuous variables and categorical variables; Q, quintile.

### 3.2 Association between ABSI, WWI, BRI, WHtR, NHHR, RFM, BMI, and WC and infertility

Logistic regression results for the association between ABSI, WWI, BRI, WHtR, NHHR, RFM, BMI, and WC and the risk of infertility are shown in **Table 2**. Individuals in the highest quintile for ABSI, WWI, BRI, WHtR, NHHR, RFM, BMI, and WC had a higher risk of infertility compared to those in the lowest quintile. The respective adjusted OR values were1.65(95% CI, 1.14 to 2.42), 1.71(95% CI, 1.15 to 2.57), 2.09(95% CI, 1.39 to 3.19), 2.09 ( 95% CI, 1.39 to 3.19), 1.71(95% CI, 1.14 to 2.59), 2.09 (95% CI, 1.39 to 3.19), 2.10 (95% CI, 1.40 to 3.18), and 2.28 (95% CI, 1.52 to 3.47), with the *p* for trend values of 0.027, <0.001, <0.001, <0.001, 0.002, <0.001, <0.001, and <0.001after controlling for age, race, education level, smoking status, regular exercise, marital status, annual household income, alcohol intake, total energy intake, AEHI, T2DM status, cancer status, CVD status, SII, systolic blood pressure, and diastolic blood pressure.

**Table 2:** The association between obesity-related indicators and female infertility among individuals aged 20–45 by logistic regression models (N = 2,875).

	Q1	Q2	Q3	Q4	Q5	*P* trend
ABSI	<75.96	75.96-78.38	78.38-78.39	78.39-83.53	≥83.53	
Case/n	51/575	62/575	64/575	63/575	90/575	
Model1	1 (Ref.)	1.17 (0.79,1.75)	1.16 (0.78,1.72)	1.07 (0.72,1.60)	1.65 (1.14,2.40)	0.023
Model2	1 (Ref.)	1.17 (0.79,1.74)	1.15 (0.78,1.72)	1.07 (0.72,1.59)	1.64 (1.13,2.39)	0.025
Model3	1 (Ref.)	1.19 (0.80,1.78)	1.18 (0.79,1.75)	1.07 (0.72,1.60)	1.65 (1.14,2.42)	0.027
WWI	<10.25	10.25-10.70	10.71-11.14	11.15-11.66	≥11.66	
Case/n	47/575	51/575	64/575	78/575	90/575	
Model1	1 (Ref.)	0.99 (0.65,1.51)	1.26 (0.84,1.90)	1.53 (1.03,2.28)	1.78 (1.21,2.64)	<0.001
Model2	1 (Ref.)	0.99 (0.65,1.50)	1.26 (0.84,1.89)	1.52 (1.03,2.27)	1.77 (1.20,2.63)	<0.001
Model3	1 (Ref.)	0.97 (0.64,1.49)	1.24 (0.83,1.86)	1.51 (1.02,2.26)	1.71 (1.15,2.57)	<0.001
BRI	<3.22	3.22-4.32	4.33-5.65	5.66-7.55	≥7.55	
Case/n	45/575	51/575	60/575	76/575	98/575	
Model1	1 (Ref.)	1.01 (0.66,1.55)	1.24 (0.82,1.88)	1.64 (1.10,2.47)	2.13 (1.45,3.16)	<0.001
Model2	1 (Ref.)	1.01 (0.66,1.55)	1.24 (0.82,1.89)	1.64 (1.10,2.47)	2.11 (1.44,3.15)	<0.001
Model3	1 (Ref.)	1.01 (0.66,1.55)	1.24 (0.82,1.89)	1.64 (1.09,2.49)	2.09 (1.39,3.19)	<0.001
WHTR	<0.49	0.49-0.54	0.55-0.60	0.61-0.69	≥0.69	
Case/n	45/575	51/575	60/575	76/575	98/575	
Model1	1 (Ref.)	1.01 (0.66,1.55)	1.24 (0.82,1.88)	1.64 (1.10,2.47)	2.13 (1.45,3.16)	<0.001
Model2	1 (Ref.)	1.01 (0.66,1.55)	1.24 (0.82,1.89)	1.64 (1.10,2.47)	2.11 (1.44,3.15)	<0.001
Model3	1 (Ref.)	1.01 (0.66,1.55)	1.24 (0.82,1.89)	1.64 (1.09,2.49)	2.09 (1.39,3.19)	<0.001
NHHR	<1.47	1.47-19.1	1.92-2.42	2.43-3.20	≥3.20	
Case/n	42/573	53/577	70/574	83/576	82/575	
Model1	1 (Ref.)	1.25 (0.82,1.92)	1.71 (1.14,2.59)	1.98 (1.33,2.97)	1.80 (1.21,2.71)	<0.001
Model2	1 (Ref.)	1.25 (0.82,1.92)	1.70 (1.14,2.58)	1.96 (1.32,2.95)	1.79 (1.20,2.70)	<0.001
Model3	1 (Ref.)	1.25 (0.82,1.93)	1.72 (1.15,2.60)	1.92 (1.28,2.90)	1.71 (1.14,2.59)	0.002
RFM	<35.38	35.38-39.57	39.58-43.22	43.23-46.90	≥46.90	
Case/n	45/575	51/575	60/575	76/575	98/575	
Model1	1 (Ref.)	1.01 (0.66,1.55)	1.24 (0.82,1.88)	1.64 (1.10,2.47)	2.13 (1.45,3.16)	<0.001
Model2	1 (Ref.)	1.01 (0.66,1.55)	1.24 (0.82,1.89)	1.64 (1.10,2.47)	2.11 (1.44,3.15)	<0.001
Model3	1 (Ref.)	1.01 (0.66,1.55)	1.24 (0.82,1.89)	1.64 (1.09,2.49)	2.09 (1.39,3.19)	<0.001
BMI	<22.30	22.30-25.99	26.00-30.19	30.20-35.89	≥35.89	
Case/n	47/565	56/576	50/581	76/573	101/580	
Model1	1 (Ref.)	1.07 (0.71,1.63)	0.95 (0.62,1.46)	1.55 (1.04,2.31)	2.12 (1.46,3.14)	<0.001
Model2	1 (Ref.)	1.08 (0.71,1.63)	0.95 (0.62,1.47)	1.54 (1.04,2.31)	2.12 (1.45,3.13)	<0.001
Model3	1 (Ref.)	1.08 (0.71,1.63)	0.96 (0.62,1.48)	1.53 (1.02,2.30)	2.10 (1.40,3.18)	<0.001
WC	<79.70	79.70-88.39	88.40-97.79	97.80-110.82	≥110.82	
Case/n	45/574	45/574	65/574	71/578	104/575	
Model1	1 (Ref.)	0.87 (0.56,1.35)	1.33 (0.88,2.01)	1.44 (0.96,2.18)	2.27 (1.55,3.37)	<0.001
Model2	1 (Ref.)	0.88 (0.57,1.36)	1.33 (0.88,2.01)	1.44 (0.96,2.18)	2.26 (1.54,3.35)	<0.001
Model3	1 (Ref.)	0.86 (0.55,1.34)	1.35 (0.89,2.05)	1.44 (0.96,2.19)	2.28 (1.52,3.47)	<0.001

Model 1 was adjusted by age, race, education level, smoking status, moderate physical activity, marital status, annual household income and alcohol intake; Model 2 was further adjusted by total energy intake and AEHI; Model 3 was further adjusted by T2DM status, cancer status, CVD status, SII, systolic blood pressure and diastolic blood pressure; Case/N, number of case subjects/total; Q, quintile.

### 3.3 RCS analysis investigating the association between ABSI, WWI, BRI, WHtR, NHHR, RFM, BMI, and WC and infertility

**Figure 1:**
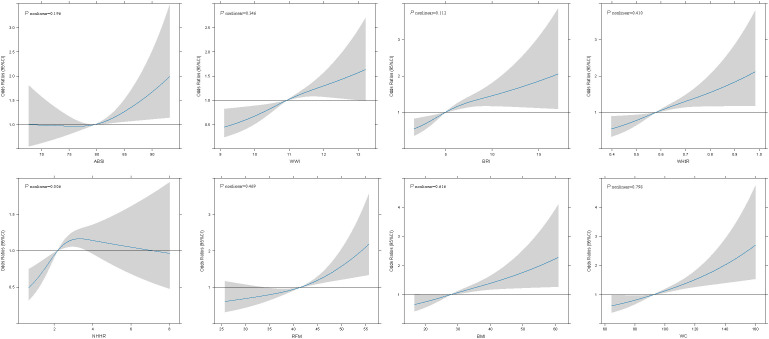
Associations between obesity-related indicators and female infertility were evaluated by RCS after adjustment for age, race, education level, smoking status, moderate physical activity, marital status, annual household income, alcohol intake, total energy intake, AEHI, T2DM status, cancer status, CVD status, SII, systolic blood pressure and diastolic blood pressure. The solid black lines correspond to the central estimates, and the gray-shaded regions indicate the 95% confidence intervals.

RCS curve was utilized to explore and visualize the associations between ABSI, WWI, BRI, WHtR, NHHR, RFM, BMI, and WC and infertility after adjusting for all covariates in the master analytical model 3 above (**Figure 1**). The research results indicated that a linear correlation was present between ABSI, WWI, BRI, WHtR, RFM, BMI, and WC and infertility (*P*_nonlinear_ > 0.05). However, a nonlinear association was observed between NHHR and infertility (*P*_nonlinear_ = 0.006).

### 3.4 ROC curves for ABSI, WWI, BRI, WHtR, NHHR, RFM, BMI, and WC and infertility

**Figure 2:**
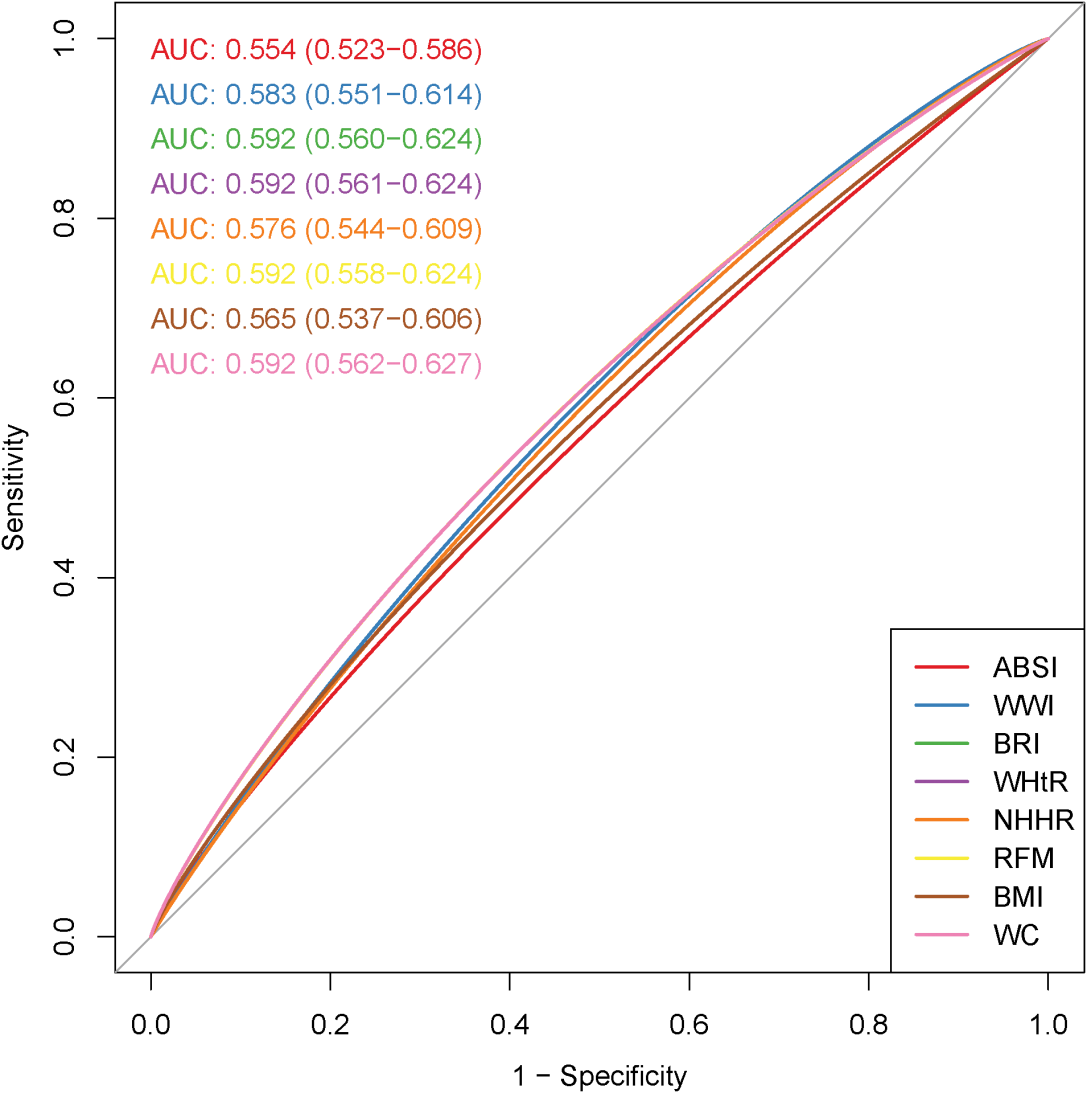
Receiver operating characteristic (ROC) curves of obesity-related indicators in relation to female infertility.

**Figure 2** demonstrates the diagnostic effects of ABSI, WWI, BRI, WHtR, NHHR, RFM, BMI, and WC on infertility. The ROC curve shows that BRI, WHtR, RFM, and WC had the highest diagnostic efficacy for infertility (AUC: 0.592, 95% CI 0.560–0.624), followed by WWI (AUC: 0.583, 95% CI 0.551–0.614) and NHHR (AUC: 0.576, 95% CI 0.544–0.609).

### 3.5 Sensitivity analysis

When infertility was determined solely based on the question "Have you ever attempted to become pregnant over at least a year, without becoming pregnant?", individuals in the highest quintile for ABSI, WWI, BRI, WHtR, NHHR, RFM, BMI, and WC had a higher risk of infertility compared to those in the lowest quintile. The respective adjusted OR values were 1.76(95% CI, 1.23 to 2.54), 1.74(95% CI, 1.19 to 2.57), 2.11(95% CI, 1.43 to 3.15), 2.11(95% CI, 1.43 to 3.15), 1.67(95% CI, 1.14 to 2.47), 2.11(95% CI, 1.43 to 3.15), 2.17(95% CI, 1.48 to 3.22), and 2.33(95% CI, 1.57 to 3.48), and with the p for trend values of <0.05. Among people aged 20-35, the respective adjusted OR values were 2.15(95% CI,1.23 to 3.87), 3.09(95% CI,1.65 to 6.10), 3.02(95% CI,1.60 to 5.98), 3.02(95% CI,1.60 to 5.98), 1.87(95% CI,1.05 to 3.44), 3.02(95% CI,1.60 to 5.98), 2.55(95% CI,1.38 to 4.78), and 2.81(95% CI,1.50 to 5.50), and with the *p* for trend values of <0.05.

**Figure 3:**
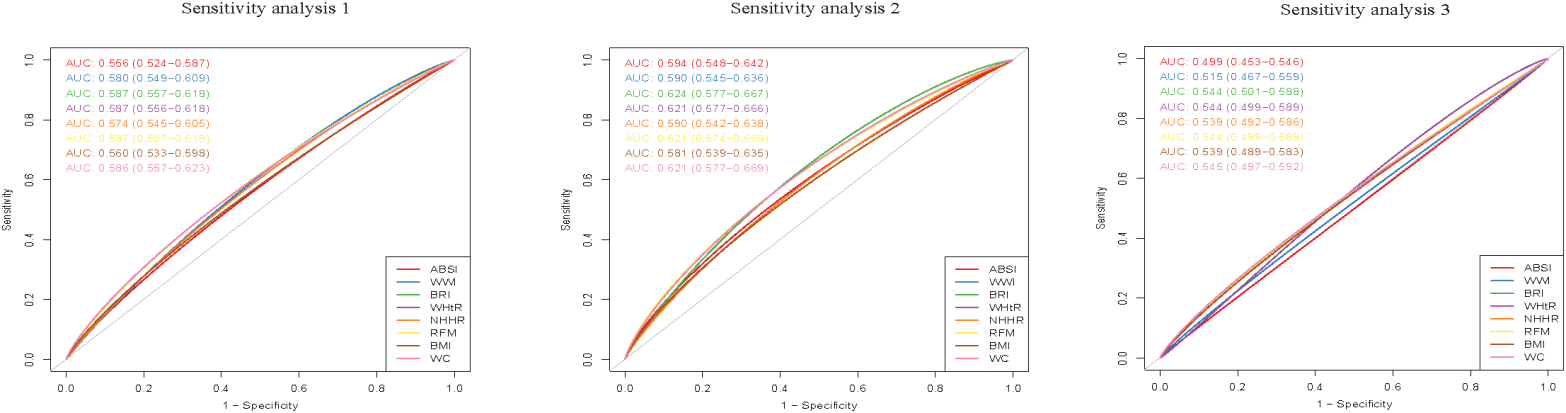
Receiver operating characteristic (ROC) curves of obesity-related indicators in relation to female infertility. Sensitivity analysis 1:Infertility was determined solely through the single question: "Have you ever attempted to become pregnant over at least a year, without becoming pregnant?". Sensitivity analysis 2:Population aged 20-35. Sensitivity analysis 3:Population aged 36-45.

However, no statistical significance was found between ABSI, WWI, and WC and infertility in the population aged 36–45 years. The respective adjusted OR values were 0.98(95% CI, 0.59 to 1.63), 1.20 (95% CI, 0.72 to 2.01), and 1.49 (95% CI, 0.88 to 2.56), with the *p* for trend values of 0.949, 0.279, and 0.073. Detailed results are provided in **Supplementary Tables 1**–**3**. In addition, BRI had relatively high diagnostic efficacy for infertility among people aged 20-35 (AUC: 0.624, 95% CI 0.577–0.667), followed by WHtR, RFM, and WC (AUC: 0.621, 95% CI 0.577–0.669), and the detailed results were presented in **Figure 3**.

## 4 Discussion

To the best of our knowledge, this is the first study to systematically explore the association between obesity-related indicators and female infertility. The study findings revealed a notable link between ABSI, WWI, BRI, WHtR, NHHR, RFM, BMI, and WC and infertility after adjusting for a series of confounding factors. The WHtR, RFM, WC, and BRI are superior to BMI in predicting and diagnosing infertility, particularly among individuals aged 20–35 years. Thus, they can serve as potential indicators for infertility prevention and intervention. Managing central obesity may help to reduce the prevalence of infertility.

In the general population, the ROC curve shows that BRI, WHtR, RFM, and WC had relatively high diagnostic efficacy for infertility (AUC: 0.592, 95% CI 0.560–0.624). However, their diagnostic utility remains limited. Among people aged 20-35, compared with other obesity-related indicators, BRI had relatively higher diagnostic efficacy for infertility (AUC: 0.624, 95% CI 0.577–0.667). Notably, the performance of BRI in diagnosing infertility was significantly better than that in the general population. This underscores the importance of focusing on the diagnostic capability of BRI in infertility in our subsequent research.

The specific mechanism that leads to the incidence of female infertility remains unclear. BMI, which relies solely on height and weight, is often used as a common indicator to determine if someone is overweight or obese, and obesity is a risk factor for infertility. Previous research has shown that excessive fat tissue can disrupt hormonal balance, thereby leading to irregularities in ovulation and menstrual cycles (22). Higher waist measurement values can indicate an excessive fat tissue deposition in the epigastrium (23). A series of indicators, including BRI, WHtR, RFM, and WC, are a new type of obesity measurement indices that take WC measurements into full consideration. They can effectively assess visceral fat distribution among individuals, providing advantages over the traditional BMI (24). In this study, we found that BRI, WHtR, RFM, and WC demonstrated significantly higher diagnostic efficacy for infertility than BMI. Previous studies have indicated BMI may not accurately predict infertility prevalence due to its limitations in distinguishing between fat and muscle mass, and not considering fat distribution, particularly in cases of abdominal obesity (8). This study has confirmed the findings of previous research. It also suggests that in the process of clinical treatment or prevention of infertility, we should place significant emphasis on the impact of central obesity on infertility. Meanwhile, we should adopt effective measures to prevent and control the occurrence of central obesity, so as to reduce the risk of infertility.

Insulin resistance (IR) may provide a plausible explanation for the present study observations. The impact of IR on reproductive function is currently receiving increasing attention. Previous studies have shown that obesity, especially abdominal obesity, can lead to IR (25). Indeed, IR is closely associated with the development of multiple metabolic disorders, including T2DM, hypertension, atherosclerosis, and polycystic ovary syndrome (PCOS). PCOS is the most common cause of anovulatory infertility (26). Moreover, IR does not only heighten the risk of infertility in women with PCOS (27), but also raises the chances of infertility in non-PCOS women of reproductive age, especially those with irregular menstruation (28).

Dysbiosis of the gut microbiota may directly or indirectly contribute to the pathogenesis of these infertility disorders. Obesity can lead to gut microbiota disorders or promote intestinal stem cell proliferation (29). The intestinal microbiota regulate estrogen metabolism through the estrogen-gut axis and estrogen metabolites (estrobolome). Dysfunctions in these mechanisms may lead to gynecological disorders, such as endometriosis, infertility, chronic pelvic pain, and dysmenorrhea (30). In addition, dysbiosis of gut microbiota may induce systemic inflammation and interfere with estrogen metabolism and receptor activation in estrogen-regulated organs, influencing neurocognition, metabolism, and the onset of gynecologic diseases and infertility (31).Together, obesity and excess fat accumulation may lead to gut microbiome disruptions, which, in turn, can cause psychological issues, such as low self-esteem, depression, and anxiety, among women via metabolic products, ultimately impacting their fertility (32, 33).

Higher BRI, WHtR, RFM, and WC values indicate more abdominal fat, which can lead to health issues. Abdominal fat deposition can lead to low-grade inflammation through the action of cytokines and adipokines (34). Visceral adipose tissue is active endocrine tissue that is capable of producing inflammatory mediators, such as tumor necrosis factor α and interleukin-6. Excessive release of these mediators may trigger a chronic inflammatory response, causing damage to vascular endothelial cells and decreasing endometrial receptivity (35, 36). Fat tissue serves as a source of lipotoxic danger signals that trigger inflammation, causing a decrease in T lymphocyte production. A recent study indicated that a reduction in T cell numbers may result in enhanced immune cell activity and inflammatory responses, potentially leading to infertility (37, 38).

Systemic oxidative stress is positively correlated with visceral fat accumulation (39). The molecular mechanism underlying infertility induced by central obesity may be attributed to the impact of oxidative stress on oocytes, leading to infertility. Excessive ROS production damages the sperm membrane, proteins, and DNA, impairing sperm motility, viability, and the ability to fertilize an oocyte (40). Oxidative stress induces antral follicle atresia in animals, and FOXO1 is a key regulatory factor of oxidative stress that triggers follicular granulosa cell apoptosis (41). Meanwhile, oxidative stress triggers lipid peroxidation. The lipid peroxidation in the plasma membrane and the disruption of Ca²^+^ homeostasis damage the fluidity of the oocyte membrane, thereby impeding the fusion process with sperm (42).

The present study has several strengths. First, it is the first to systematically explore the relationship between obesity-related indicators and infertility risk using large-sample data. It emphasizes the relatively higher diagnostic efficacy of BRI, WHtR, RFM, and WC in predicting infertility among individuals aged 20 - 35. When compared with BMI, these indices show a more pronounced advantage in this regard. Second, NHANES is a nationally representative database based on a probability sample survey design in the United States that provides the most comprehensive and authoritative information on demographics and infertility, along with a detailed evaluation of lifestyle factors. Third, stratified analyses were conducted across different age groups, revealing significant relationships between obesity-related indicators and infertility in individuals aged 20–35 years.

However, several limitations should also be considered. First, the NHANES data are cross-sectional, which prevents us from inferring causal relationships between obesity-related indicators and infertility. Future studies may leverage Mendelian randomization or cohort studies to further explore the causal link between the two. Second, the outcome indicator of infertility is based on self - reporting. Thus, there may be recall bias as well as social desirability bias resulting from societal expectations, which may have inflated the prevalence estimate. Additionally, during the process of self - disclosure, respondents may classify information based on their own subjective feelings or experiences, leading to misclassification. Third, the study was conducted using solely the data from the United States, and although multiple ethnicities were included, the generalizability of the findings to the general population requires further confirmation through large-scale prospective cohort studies. Last, despite adjusting for a wide range of major confounding factors during the analysis, any associations between obesity-related indicators and infertility may still be influenced by other unobserved or unknown confounding factors, such as unavailability of detailed information on spermiograms, PCOS and endometriosis, which may impact the study results. PCOS can lead to elevated androgen levels (hyperandrogenism), the development of ovarian cysts or ovarian enlargement, ovulatory dysfunction (such as anovulation), and irregular menstrual cycles or amenorrhea. On the other hand, endometriosis is associated with the release of inflammatory mediators in the peritoneal fluid, which create an altered microenvironment that subsequently results in poor oocyte/embryo quality and reduced implantation rates.

## 5 Conclusions

Obesity-related indicators (ABSI, WWI, BRI, WHtR, NHHR, RFM, BMI, and WC) are associated with the incidence of infertility. The WHtR, RFM, WC, and BRI are superior to BMI in predicting and diagnosing infertility, particularly among individuals aged 20–35 years. Consequently, these indices show promise as more effective tools for identifying populations at an early risk of infertility. To confirm these findings, future studies, such as Mendelian randomization or cohort studies, are warranted.

## Data Availability

The original contributions presented in the study are included in the article/**Supplementary Material**. Further inquiries can be directed to the corresponding author.
