# Supplementary material for: The association between obesity-related indicators and female infertility: the United States National Health and Nutrition Examination Survey, 2013–2018
Source: Front Endocrinol (Lausanne). 2025 Jun 23;16:1588965. doi: 10.3389/fendo.2025.1588965 (PMC12229851; doi:10.3389/fendo.2025.1588965)
Supplement: Supplementary file 1 [file Table1.docx]

**Supplement Table 1.** The association between obesity-related indicators and female infertility among individuals aged 20-45 by logistic regression models (N = 2,875)

|  | **Q1** | **Q2** | **Q3** | **Q4** | **Q5** | ***P* trend** |
| --- | --- | --- | --- | --- | --- | --- |
| **ABSI** | <75.96 | 75.96-78.38 | 78.38-78.39 | 78.39-83.53 | ≥83.53 |  |
| Case/n | 55/575 | 69/575 | 74/575 | 71/575 | 100/575 |  |
| Model1 | 1(Ref.) | 1.22(0.84,1.79) | 1.26(0.87,1.85) | 1.14(0.78,1.67) | 1.73(1.21,2.49) | 0.009 |
| Model2 | 1(Ref.) | 1.22(0.84,1.79) | 1.26(0.87,1.85) | 1.14(0.78,1.66) | 1.73(1.21,2.49) | 0.009 |
| Model3 | 1(Ref.) | 1.24(0.85,1.82) | 1.29(0.88,1.88) | 1.14(0.78,1.67) | 1.76(1.23,2.54) | 0.009 |
| **WWI** | <10.25 | 10.25-10.70 | 10.71-11.14 | 11.15-11.66 | ≥11.66 |  |
| Case/n | 53/575 | 58/575 | 72/575 | 87/575 | 99/575 |  |
| Model1 | 1(Ref.) | 1.00(0.67,1.49) | 1.27(0.87,1.88) | 1.54(1.06,2.24) | 1.77(1.22,2.57) | <0.001 |
| Model2 | 1(Ref.) | 1.00(0.67,1.49) | 1.28(0.87,1.88) | 1.54(1.06,2.25) | 1.77(1.22,2.58) | <0.001 |
| Model3 | 1(Ref.) | 1.00(0.67,1.49) | 1.26(0.86,1.85) | 1.54(1.06,2.26) | 1.74(1.19,2.57) | <0.001 |
| **BRI** | <3.22 | 3.22-4.32 | 4.33-5.65 | 5.66-7.55 | ≥7.55 |  |
| Case/n | 51/575 | 59/575 | 69/575 | 83/575 | 107/575 |  |
| Model1 | 1(Ref.) | 1.04(0.70,1.55) | 1.27(0.86,1.89) | 1.60(1.09,2.35) | 2.08(1.44,3.03) | <0.001 |
| Model2 | 1(Ref.) | 1.04(0.70,1.56) | 1.27(0.86,1.89) | 1.60(1.09,2.36) | 2.08(1.44,3.04) | <0.001 |
| Model3 | 1(Ref.) | 1.04(0.70,1.56) | 1.28(0.87,1.90) | 1.62(1.10,2.41) | 2.11(1.43,3.15) | <0.001 |
| **WHtR** | <0.49 | 0.49-0.54 | 0.55-0.60 | 0.61-0.69 | ≥0.69 |  |
| Case/n | 51/575 | 59/575 | 69/575 | 83/575 | 107/575 |  |
| Model1 | 1(Ref.) | 1.04(0.70,1.55) | 1.27(0.86,1.89) | 1.60(1.09,2.35) | 2.08(1.44,3.03) | <0.001 |
| Model2 | 1(Ref.) | 1.04(0.70,1.56) | 1.27(0.86,1.89) | 1.60(1.09,2.36) | 2.08(1.44,3.04) | <0.001 |
| Model3 | 1(Ref.) | 1.04(0.70,1.56) | 1.28(0.87,1.90) | 1.62(1.10,2.41) | 2.11(1.43,3.15) | <0.001 |
| **NHHR** | <1.47 | 1.47-19.1 | 1.92-2.42 | 2.43-3.20 | ≥3.20 |  |
| Case/n | 50/573 | 57/577 | 78/574 | 92/576 | 92/575 |  |
| Model1 | 1(Ref.) | 1.12(0.75,1.68) | 1.61(1.10,2.36) | 1.86(1.28,2.72) | 1.72(1.18,2.52) | <0.001 |
| Model2 | 1(Ref.) | 1.12(0.75,1.69) | 1.61(1.10,2.37) | 1.86(1.28,2.73) | 1.73(1.19,2.54) | <0.001 |
| Model3 | 1(Ref.) | 1.13(0.75,1.70) | 1.64(1.12,2.41) | 1.83(1.26,2.70) | 1.67(1.14,2.47) | <0.001 |
| **RFM** | <35.38 | 35.38-39.57 | 39.58-43.22 | 43.23-46.90 | ≥46.90 |  |
| Case/n | 51/575 | 59/575 | 69/575 | 83/575 | 107/575 |  |
| Model1 | 1(Ref.) | 1.04(0.70,1.55) | 1.27(0.86,1.89) | 1.60(1.09,2.35) | 2.08(1.44,3.03) | <0.001 |
| Model2 | 1(Ref.) | 1.04(0.70,1.56) | 1.27(0.86,1.89) | 1.60(1.09,2.36) | 2.08(1.44,3.04) | <0.001 |
| Model3 | 1(Ref.) | 1.04(0.70,1.56) | 1.28(0.87,1.90) | 1.62(1.10,2.41) | 2.11(1.43,3.15) | <0.001 |
| **BMI** | <22.30 | 22.30-25.99 | 26.00-30.19 | 30.20-35.89 | ≥35.90 |  |
| Case/n | 53/565 | 66/576 | 57/581 | 81/573 | 112/580 |  |
| Model1 | 1(Ref.) | 1.14(0.77,1.68) | 0.97(0.65,1.45) | 1.47(1.00,2.15) | 2.12(1.48,3.07) | <0.001 |
| Model2 | 1(Ref.) | 1.14(0.77,1.68) | 0.97(0.65,1.45) | 1.47(1.00,2.16) | 2.13(1.48,3.08) | <0.001 |
| Model3 | 1(Ref.) | 1.14(0.77,1.69) | 0.99(0.66,1.48) | 1.47(1.00,2.18) | 2.17(1.48,3.22) | <0.001 |
| **WC** | <79.70 | 79.70-88.39 | 88.4-97.79 | 97.80-110.82 | ≥110.82 |  |
| Case/n | 50/574 | 53/574 | 75/574 | 78/578 | 113/575 |  |
| Model1 | 1(Ref.) | 0.93(0.62,1.41) | 1.40(0.95,2.08) | 1.44(0.98,2.14) | 2.25(1.56,3.28) | <0.001 |
| Model2 | 1(Ref.) | 0.93(0.62,1.41) | 1.40(0.95,2.08) | 1.44(0.98,2.14) | 2.25(1.56,3.29) | <0.001 |
| Model3 | 1(Ref.) | 0.92(0.61,1.40) | 1.43(0.97,2.13) | 1.47(0.99,2.19) | 2.33(1.57,3.48) | <0.001 |

Model 1 was adjusted by age, race, education level, smoking status, moderate physical activity, marital status, annual household income and alcohol intake; Model 2 was further adjusted by total energy intake and AEHI; Model 3 was further adjusted by T2DM status, cancer status, CVD status, SII, systolic blood pressure and diastolic blood pressure; Case/N, number of case subjects/total; Q, quintile.

**Supplement Table 2.** The association between obesity-related indicators and female infertility among individuals aged 20-35 by logistic regression models (N = 1,675)

|  | Q1 | Q2 | Q3 | Q4 | Q5 | *P* trend |
| --- | --- | --- | --- | --- | --- | --- |
| **ABSI** | <75.56 | 75.56-77.98 | 77.99-80.31 | 80.32-83.23 | ≥83.24 |  |
| Case/n | 20/335 | 21/335 | 31/335 | 26/335 | 51/335 |  |
| Model1 | 1(Ref) | 0.94(0.50,1.80) | 1.42(0.79,2.61) | 1.09(0.59,2.04) | 2.23(1.30,3.96) | 0.002 |
| Model2 | 1(Ref) | 0.94(0.49,1.79) | 1.41(0.78,2.59) | 1.07(0.58,2.00) | 2.19(1.27,3.89) | 0.003 |
| Model3 | 1(Ref) | 0.95(0.50,1.83) | 1.42(0.79,2.62) | 1.10(0.59,2.06) | 2.15(1.23,3.87) | 0.005 |
| **WWI** | <10.15 | 10.15-10.60 | 10.61-11.04 | 11.05-11.58 | ≥11.59 |  |
| Case/n | 14/335 | 16/335 | 32/335 | 36/335 | 51/335 |  |
| Model1 | 1(Ref) | 1.05(0.50,2.23) | 2.14(1.13,4.25) | 2.10(1.12,4.17) | 3.21(1.75,6.22) | <0.001 |
| Model2 | 1(Ref) | 1.05(0.50,2.22) | 2.11(1.12,4.20) | 2.10(1.12,4.17) | 3.11(1.70,6.05) | <0.001 |
| Model3 | 1(Ref) | 1.05(0.50,2.23) | 2.07(1.09,4.12) | 2.02(1.07,4.04) | 3.09(1.65,6.10) | <0.001 |
| **BRI** | <2.96 | 2.96-4.02 | 4.03-5.29 | 5.30-7.27 | ≥7.28 |  |
| Case/n | 15/335 | 17/335 | 24/335 | 42/335 | 51/335 |  |
| Model1 | 1(Ref) | 0.99(0.48,2.05) | 1.35(0.69,2.71) | 2.38(1.29,4.59) | 3.06(1.69,5.84) | <0.001 |
| Model2 | 1(Ref) | 0.98(0.48,2.04) | 1.35(0.69,2.71) | 2.35(1.27,4.54) | 2.99(1.64,5.72) | <0.001 |
| Model3 | 1(Ref) | 0.99(0.48,2.06) | 1.36(0.69,2.74) | 2.37(1.27,4.63) | 3.02(1.60,5.98) | <0.001 |
| **WHtR** | <0.48 | 0.48-0.52 | 0.53-0.58 | 0.59-0.67 | ≥0.68 |  |
| Case/n | 17/325 | 24/342 | 21/333 | 35/339 | 52/336 |  |
| Model1 | 1(Ref) | 0.99(0.48,2.05) | 1.35(0.69,2.71) | 2.38(1.29,4.59) | 3.06(1.69,5.84) | <0.001 |
| Model2 | 1(Ref) | 0.98(0.48,2.04) | 1.35(0.69,2.71) | 2.35(1.27,4.54) | 2.99(1.64,5.72) | <0.001 |
| Model3 | 1(Ref) | 0.99(0.48,2.06) | 1.36(0.69,2.74) | 2.37(1.27,4.63) | 3.02(1.60,5.98) | <0.001 |
| **NHHR** | <1.41 | 1.41-1.83 | 1.84-2.29 | 2.30-2.99 | ≥3.00 |  |
| Case/n | 19/335 | 25/335 | 22/335 | 43/338 | 40/332 |  |
| Model1 | 1(Ref) | 1.35(0.72,2.54) | 1.11(0.58,2.13) | 2.03(1.15,3.70) | 1.96(1.12,3.56) | 0.005 |
| Model2 | 1(Ref) | 1.31(0.70,2.48) | 1.07(0.56,2.06) | 1.97(1.11,3.60) | 1.88(1.07,3.43) | 0.006 |
| Model3 | 1(Ref) | 1.27(0.68,2.41) | 1.04(0.55,2.01) | 1.70(0.95,3.13) | 1.87(1.05,3.44) | 0.016 |
| **RFM** | <34.17 | 34.17-35.57 | 38.58-42.33 | 42.34-46.43 | ≥46.44 |  |
| Case/n | 335/325 | 335/342 | 335/333 | 335/339 | 335/336 |  |
| Model1 | 1(Ref) | 0.99(0.48,2.05) | 1.35(0.69,2.71) | 2.38(1.29,4.59) | 3.06(1.69,5.84) | <0.001 |
| Model2 | 1(Ref) | 0.98(0.48,2.04) | 1.35(0.69,2.71) | 2.35(1.27,4.54) | 2.99(1.64,5.72) | <0.001 |
| Model3 | 1(Ref) | 0.99(0.48,2.06) | 1.36(0.69,2.74) | 2.37(1.27,4.63) | 3.02(1.60,5.98) | <0.001 |
| **BMI** | <21.8 | 21.8-25.19 | 25.20-28.99 | 29.00-34.99 | ≥35 |  |
| Case/n | 17/325 | 24/342 | 21/333 | 35/339 | 52/336 |  |
| Model1 | 1(Ref) | 1.19(0.63,2.32) | 0.96(0.49,1.91) | 1.75(0.96,3.31) | 2.62(1.48,4.83) | <0.001 |
| Model2 | 1(Ref) | 1.21(0.63,2.35) | 0.98(0.50,1.94) | 1.75(0.96,3.30) | 2.59(1.47,4.79) | <0.001 |
| Model3 | 1(Ref) | 1.22(0.64,2.38) | 1.00(0.51,1.99) | 1.76(0.96,3.36) | 2.55(1.38,4.87) | <0.001 |
| **WC** | <77.68 | 77.68-86.19 | 86.20-95.39 | 95.40-108.79 | ≥108.8 |  |
| Case/n | 16/335 | 16/333 | 27/335 | 37/336 | 53/336 |  |
| Model1 | 1(Ref) | 0.84(0.40,1.73) | 1.41(0.74,2.76) | 1.93(1.05,3.68) | 2.86(1.60,5.37) | <0.001 |
| Model2 | 1(Ref) | 0.83(0.40,1.72) | 1.40(0.74,2.74) | 1.93(1.05,3.69) | 2.80(1.57,5.26) | <0.001 |
| Model3 | 1(Ref) | 0.84(0.41,1.74) | 1.42(0.74,2.79) | 1.98(1.07,3.80) | 2.81(1.50,5.50) | <0.001 |

Model 1 was adjusted by age, race, education level, smoking status, moderate physical activity, marital status, annual household income and alcohol intake; Model 2 was further adjusted by total energy intake and AEHI; Model 3 was further adjusted by T2DM status, cancer status, CVD status, SII, systolic blood pressure and diastolic blood pressure; Case/N, number of case subjects/total; Q, quintile.

**Supplement Table 3.** The association between obesity-related indicators and female infertility among individuals aged 36-45 by logistic regression models (N = 1,200)

|  | Q1 | Q2 | Q3 | Q4 | Q5 | *P* trend |
| --- | --- | --- | --- | --- | --- | --- |
| **ABSI** | <76.61 | 76.61-79.01 | 79.02-81.11 | 81.12-83.76 | ≥83.77 |  |
| Case/n | 38/240 | 32/240 | 39/240 | 36/240 | 36/240 |  |
| Model1 | 1(Ref) | 0.81(0.48,1.36) | 0.99(0.61,1.63) | 0.90(0.54,1.49) | 0.95(0.57,1.57) | 0.987 |
| Model2 | 1(Ref) | 0.82(0.49,1.37) | 1.00(0.61,1.64) | 0.90(0.55,1.50) | 0.95(0.58,1.58) | 0.996 |
| Model3 | 1(Ref) | 0.85(0.50,1.43) | 1.01(0.62,1.67) | 0.93(0.56,1.54) | 0.98(0.59,1.63) | 0.949 |
| **WWI** | <10.42 | 10.42-10.84 | 10.85-11.28 | 11.29-11.77 | ≥11.78 |  |
| Case/n | 36/240 | 34/240 | 32/240 | 41/240 | 38/240 |  |
| Model1 | 1(Ref) | 0.96(0.57,1.60) | 0.93(0.55,1.57) | 1.32(0.80,2.18) | 1.19(0.71,2.00) | 0.251 |
| Model2 | 1(Ref) | 0.95(0.57,1.60) | 0.93(0.55,1.57) | 1.32(0.80,2.19) | 1.20(0.72,2.01) | 0.240 |
| Model3 | 1(Ref) | 0.94(0.56,1.58) | 0.95(0.56,1.60) | 1.34(0.81,2.24) | 1.17(0.68,2.01) | 0.279 |
| **BRI** | <3.6 | 3.60-4.74 | 4.75-6.07 | 6.08-7.93 | ≥7.94 |  |
| Case/n | 32/240 | 35/240 | 32/240 | 39/240 | 43/240 |  |
| Model1 | 1(Ref) | 1.23(0.73,2.09) | 1.18(0.69,2.04) | 1.60(0.95,2.73) | 1.67(1.00,2.82) | 0.030 |
| Model2 | 1(Ref) | 1.24(0.73,2.10) | 1.19(0.69,2.04) | 1.61(0.95,2.74) | 1.70(1.01,2.88) | 0.027 |
| Model3 | 1(Ref) | 1.26(0.74,2.15) | 1.22(0.70,2.11) | 1.60(0.93,2.76) | 1.73(0.99,3.05) | 0.041 |
| **WHtR** | <0.51 | 0.51-0.56 | 0.57-0.62 | 0.63-0.69 | ≥0.7 |  |
| Case/n | 32/240 | 35/240 | 32/240 | 39/240 | 43/240 |  |
| Model1 | 1(Ref) | 1.23(0.73,2.09) | 1.18(0.69,2.04) | 1.60(0.95,2.73) | 1.67(1.00,2.82) | 0.030 |
| Model2 | 1(Ref) | 1.24(0.73,2.10) | 1.19(0.69,2.04) | 1.61(0.95,2.74) | 1.70(1.01,2.88) | 0.027 |
| Model3 | 1(Ref) | 1.26(0.74,2.15) | 1.22(0.70,2.11) | 1.60(0.93,2.76) | 1.73(0.99,3.05) | 0.041 |
| **NHHR** | <1.55 | 1.55-2.08 | 2.09-2.64 | 2.65-3.43 | ≥3.44 |  |
| Case/n | 24/240 | 36/240 | 44/240 | 38/240 | 39/240 |  |
| Model1 | 1(Ref) | 1.58(0.91,2.79) | 2.12(1.25,3.69) | 1.87(1.07,3.30) | 1.90(1.10,3.36) | 0.025 |
| Model2 | 1(Ref) | 1.58(0.91,2.79) | 2.14(1.26,3.71) | 1.90(1.09,3.36) | 1.93(1.11,3.42) | 0.021 |
| Model3 | 1(Ref) | 1.66(0.95,2.94) | 2.23(1.31,3.90) | 1.93(1.10,3.45) | 1.94(1.10,3.49) | 0.026 |
| **RFM** | <36.99 | 36.99-40.84 | 40.85-44.14 | 44.15-44.14 | ≥47.51 |  |
| Case/n | 32/240 | 35/240 | 32/240 | 39/240 | 43/240 |  |
| Model1 | 1(Ref) | 1.23(0.73,2.09) | 1.18(0.69,2.04) | 1.60(0.95,2.73) | 1.67(1.00,2.82) | 0.030 |
| Model2 | 1(Ref) | 1.24(0.73,2.10) | 1.19(0.69,2.04) | 1.61(0.95,2.74) | 1.70(1.01,2.88) | 0.027 |
| Model3 | 1(Ref) | 1.26(0.74,2.15) | 1.22(0.70,2.11) | 1.60(0.93,2.76) | 1.73(0.99,3.05) | 0.041 |
| **BMI** | <23.4 | 23.40-27.0 | 27.10-31.39 | 31.40-36.71 | ≥36.72 |  |
| Case/n | 36/236 | 29/239 | 32/243 | 40/242 | 44/240 |  |
| Model1 | 1(Ref) | 0.86(0.50,1.46) | 1.05(0.61,1.79) | 1.35(0.81,2.26) | 1.50(0.91,2.50) | 0.028 |
| Model2 | 1(Ref) | 0.86(0.50,1.46) | 1.05(0.62,1.79) | 1.36(0.81,2.27) | 1.53(0.92,2.54) | 0.026 |
| Model3 | 1(Ref) | 0.85(0.50,1.46) | 1.04(0.60,1.79) | 1.36(0.81,2.32) | 1.51(0.88,2.61) | 0.045 |
| **WC** | <82.2 | 82.20-91.3 | 91.40-101.19 | 101.20-112.51 | ≥112.52 |  |
| Case/n | 38/236 | 26/242 | 37/239 | 32/243 | 48/240 |  |
| Model1 | 1(Ref) | 0.69(0.40,1.19) | 1.12(0.67,1.87) | 0.97(0.57,1.64) | 1.48(0.91,2.43) | 0.050 |
| Model2 | 1(Ref) | 0.69(0.40,1.18) | 1.12(0.67,1.86) | 0.97(0.57,1.64) | 1.50(0.92,2.46) | 0.046 |
| Model3 | 1(Ref) | 0.67(0.38,1.16) | 1.13(0.67,1.89) | 0.94(0.54,1.61) | 1.49(0.88,2.56) | 0.073 |

Model 1 was adjusted by age, race, education level, smoking status, moderate physical activity, marital status, annual household income and alcohol intake; Model 2 was further adjusted by total energy intake and AEHI; Model 3 was further adjusted by T2DM status, cancer status, CVD status, SII, systolic blood pressure and diastolic blood pressure; Case/N, number of case subjects/total; Q, quintile.
